# Controlling X-ray beam trajectory with a flexible hollow glass fibre

**DOI:** 10.1107/S1600577513029706

**Published:** 2013-12-10

**Authors:** Yoshihito Tanaka, Takashi Nakatani, Rena Onitsuka, Kei Sawada, Isao Takahashi

**Affiliations:** a RIKEN SPring-8 Center, RIKEN, 1-1-1 Kouto, Sayo-cho, Hyogo 679-5148, Japan; b Department of Physics, School of Science and Technology, Kwansei Gakuin University, Gakuen, Sanda, Hyogo 669-1337, Japan

**Keywords:** X-ray fibre optics, beam trajectory control, absorption mapping

## Abstract

A metre-length flexible hollow glass fibre with 20 µm-bore and 1.5 mm-cladding diameters for transporting a synchrotron X-ray beam and controlling the trajectory has been examined. The large cladding diameter maintains a moderate curvature to satisfy the shallow glancing angle of total reflection. The observed transmission efficiency was more than 20% at 12.4 keV. As a demonstration, a wide-area scan of a synchrotron radiation beam was performed to identify the elements for a fixed metal film through its absorption spectra.

## Abstract 2

X-ray beam trajectory control has been performed by using a 1.5 m-long flexible hollow glass fibre. A two-dimensional scan of a synchrotron radiation beam was demonstrated for X-ray absorption mapping.

## 1. Introduction

Recent progress in synchrotron X-ray sources increasingly requests the appropriate beam handling technique for advanced and effective uses in experiments. Beam trajectory control is one of the important techniques for irradiation of a sample. Fibre optics, widely used in the visible region, are attractive for controlling the beam trajectory. X-ray beam trajectory control with fibre optics will lead to effective and advanced use of X-ray beams, such as (i) concentration of X-ray irradiation on the sample, (ii) scanning measurement for a fixed sample, (iii) beam sharing, (iv) irradiation timing control with a curved trajectory, (v) polarization rotation with a coiled fibre (Berry, 1987 ▶), and (vi) coaxial and simultaneous transport of X-ray and optical beams. The development of beam transport technology will directly offer the opportunity for X-ray pump and X-ray probe measurement, X-ray photon correlation spectroscopy, real-time X-ray computed tomography, polarization control in the soft X-ray region, as well as beam position scanning.

X-ray fibres are, however, unfamiliar in contrast to optical fibres in the visible region, and are still under development. Many attempts have been made to guide an X-ray beam with a glass capillary: a hollow glass capillary called a ‘light pipe’ (Mosher & Stephanakis, 1976 ▶) has been used chiefly for beam focusing and collimating optics (Bjeoumikhov & Bjeoumikhova, 2008 ▶; Bilderback, 2003 ▶; Snigirev *et al.*, 2007 ▶) with a tapered and bundled (poly) capillary (Bilderback & Thiel, 1995 ▶; Kumakhov, 1990 ▶), and has been applied to mapping (Bilderback *et al.*, 1994 ▶) and imaging experiments (Kruger *et al.*, 1996 ▶). Such schemes have also been examined focusing neutron (Wilkins *et al.*, 1989 ▶; Kumakhov, 2004 ▶), muon (Tomono *et al.*, 2011 ▶) and highly charged ion (Ikeda *et al.*, 2006 ▶) beams. As for longer transport of the beam, a 630 mm-long glass tube with 0.5 mm inside diameter and 6.0 mm outside diameter was utilized as an X-ray guide to transport white X-rays in the energy range from 1.5 to 50 keV (Nakazawa, 1983 ▶). A 1.6 m-long single hollow glass fibre with a gradually diminishing bore diameter also achieved beam-size compression from 470 µm to 110 µm and obtained gain for both X-ray tube and synchrotron radiation in the 8 keV to 20 keV region (Thiel *et al.*, 1992 ▶). In the soft X-ray region a similar scheme was performed with an inner metal coating and obtained almost double the transmission efficiency (Matsuura *et al.*, 2005 ▶). However, a metre-length beam transport for trajectory control has never been reported, which may be due to difficulties in curvature control and alignment for input.

In this paper we propose an X-ray fibre to transport the beam using a metre-long flexible hollow capillary with a designable trajectory, and report the performance examined with the well defined X-ray beam delivered from an undulator source. We also demonstrate the trajectory control for a parallel beam axis shift, as the beam shift is equivalent to a sample scan which is a general way for mapping at X-ray facilities (Horowitz & Howell, 1972 ▶).

## 2. Design of the X-ray fibre

**Figure 1:**
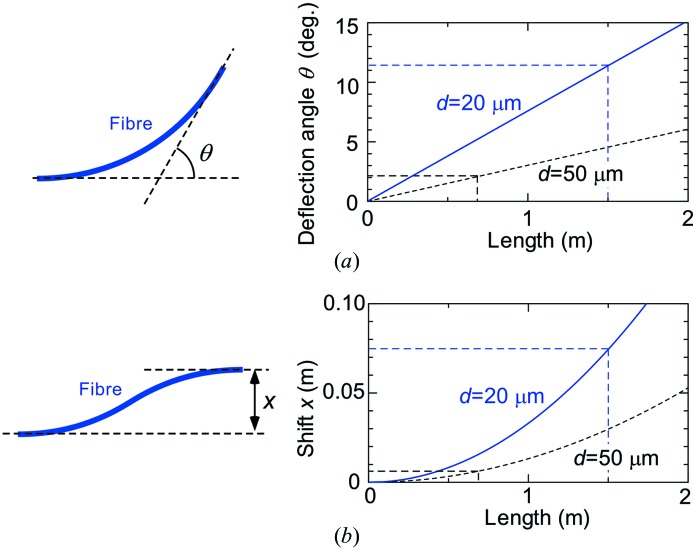
(*a*) Estimation of the available beam deflection by X-ray fibres with bore diameters of 20 µm and 50 µm, from equation (2). (*b*) Estimation of the available beam axis shift calculated from (3).

The X-ray fibre dimensions were estimated for a beam axis shift with a scan range of over 100 mm, to which we set a target because of the comparable size of an existing X-ray area detector. The design is based on the geometrical optics where an X-ray beam propagates with multiple total reflections inside a hollow glass fibre. The beam reflections are attributed to the refraction index, *n*, being slightly smaller than unity: *n* = 1 − δ − *i*β, where 1 >> δ > 0, and β is the imaginary part of the refractive index. The critical angle of total reflection, θ_c_, is given by θ_c_ ≃ (2δ)^1/2^, by using the relation 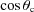 ≃ 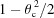 for 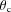 << 1 in Snell’s law. The curvature radius of the fibre, *R*, is thus limited due to the small θ_c_, as (Kumakhov, 1990 ▶; Xiao *et al.*, 1994 ▶)
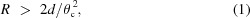
where *d* is the bore diameter. From relation (1) the beam deflection by θ satisfies
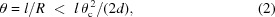
where *l* is the fibre length. For a beam axis shift, the fibre has two deflecting components with a length of *l*/2. The shift range, *x*, is then given by using the relation 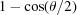 ≃ 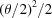, as
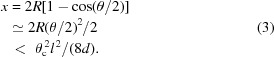
Figs. 1(*a*) and 1(*b*) ▶ show the length dependence of the maximum beam deflection and the shift for hollow fibres with bore diameters of *d* = 20 µm and 50 µm (Tanaka *et al.*, 2013 ▶). The fibres are made of borosilicate glass with θ_c_ ≃ 2.3 mrad at a photon energy of 12.4 keV. From the graph of Fig. 1(*b*) ▶, a fibre was designed to have dimensions of *d* = 20 µm and *l* = 1.5 m, to achieve a scan length of about 150 mm. We emphasize that the fibre length, *l*, is a key to achieving larger beam axis shift, because the maximum value of *x* is proportional to *l* ^2^, as seen in (3). Moreover, the optical-timing delay by curved trajectory is proportional to *l* ^3^, indicating the importance of longer transport for a tunable delay in X-ray pump–probe experiments. Another important dimension is the cladding diameter of the glass fibre, related to the flexibility. As the permissible curvature is larger than 7.6 m for *d* = 20 µm, calculated from (1), the cladding diameter was decided empirically to be 1.5 mm.

## 3. Experimental set-up

**Figure 2:**
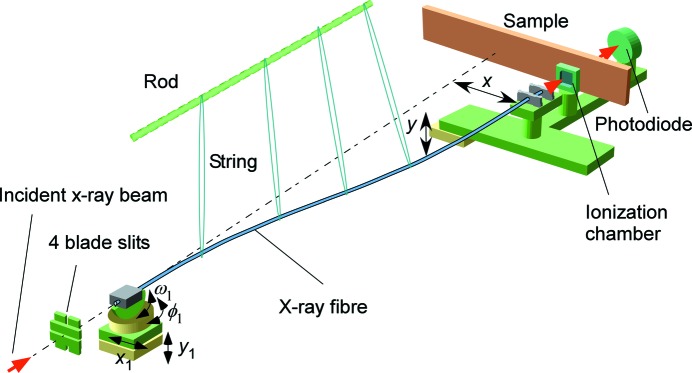
Schematic illustration of the experimental set-up for X-ray fibre control and measurement of absorption mapping.

Fig. 2 ▶ shows the equipment for X-ray fibre control and performance evaluation. The 1.5 m-long X-ray fibre manufactured by IFG was examined using a well qualified X-ray beam at undulator beamlines BL19LXU (Yabashi *et al.*, 2001 ▶) and BL29XU (Tamasaku *et al.*, 2001 ▶) at SPring-8. The experimental stations have a dimension of 5 m in the beam direction, which is sufficient for a test experiment using a 1.5 m-long X-ray fibre. The photon energy of the X-rays was tuned to 12.4 keV with a bandwidth of ∼1 eV. The beam was well collimated using a set of four-blade slits to be almost parallel in which the divergence angle is much less than 0.1 mrad.

The input coupler adjustment is dominant in the fibre alignment procedure. The X-ray fibre axis at the input side was finely adjusted to be parallel to the X-ray beam axis by using swivel, rotation and translation stages with stepper motors. Alignment of the input end so that it is on the centre position of the incident X-ray beam and parallel to the beam was conducted as follows: first, a fibre holder was finely adjusted with respect to the X-ray beam by monitoring the shadow of the holder using a flat-panel detector (Hamamatsu, C10158DK), whose area and pixel size are 100 mm × 100 mm and 50 µm, respectively; second, a capillary with *l* = 0.7 m and *d* = 50 µm was set on the holder and the same procedure as for the fibre holder was performed to obtain throughput beam on the flat panel; then, the fibre was replaced by a metre-length fibre (*l* = 1.5 m, *d* = 20 µm) and the holder was again finely adjusted to optimize the output beam property. After finishing the alignment, the flat-panel detector was replaced by a pin-photodiode attached on the end-support table for measurement of output intensity. Using the photodiode and the input-end stages, the acceptance angle was measured to be ∼0.8 mrad, which is slightly narrower than the numerical aperture [≃(2δ)^1/2^ ≃ 2.3 × 10^−3^]. This is due to absorption effects on the inside wall. The input coupler thus requires an angle tuning mechanism with precision in the milliradian range for alignment to a well collimated beam.

The X-ray fibre, owing to its flexibility, was suspended by four 300 mm-long strings so as not to hang free. The output end of the X-ray fibre was loosely supported by two sequentially aligned holders on the table so as not to apply additional force to the fibre during the position scanning. The end support table is equipped with a stepper motor system for movement in the horizontal and vertical directions for demonstration of mapping experiments.

## 4. Performance of X-ray beam transport

The performance of the X-ray beam transport and the beam axis shift with the fibre is shown here. The important parameters are the transmittance in efficiency and in flux (§4.1), the photon energy dependence (§4.2), the beam shifted area (§4.3) and the output beam profile (§4.4).

### 4.1. Transmittance in efficiency and in flux

The observed transmission efficiency was 20% at 12.4 keV at the almost straight condition of *x * ≃ 0. Here the efficiency is defined to be the ratio of the X-ray intensity with the X-ray fibre to that without the X-ray fibre, as in the previous report (Sun & Ding, 2005 ▶). The efficiency of 54% at 13 keV, reported for a 1.6 m-long tapered fused silica fibre with an inner diameter of 470–110 µm (Thiel *et al.*, 1992 ▶), is two to three times larger than our result. The smaller efficiency in the present data may be due to the difference in diameters, inner wall roughness and material.

The flux observed at the output end was up to 8 × 10^9^ photons s^−1^ at BL19LXU for *x* ≃ 0. The flux is limited by the maximum output power from a light source of the BL19LXU undulator. The fibre maintained its performance with the throughput power for hours, while the colour at the input end gradually turned black. As the beam path in the fibre was not evacuated or purged by helium in our measurement, the estimated maximum throughput flux was 1.6 × 10^10^ photons s^−1^ under the helium-purged conditions (Thiel *et al.*, 1992 ▶).

### 4.2. Photon energy dependence

**Figure 3:**
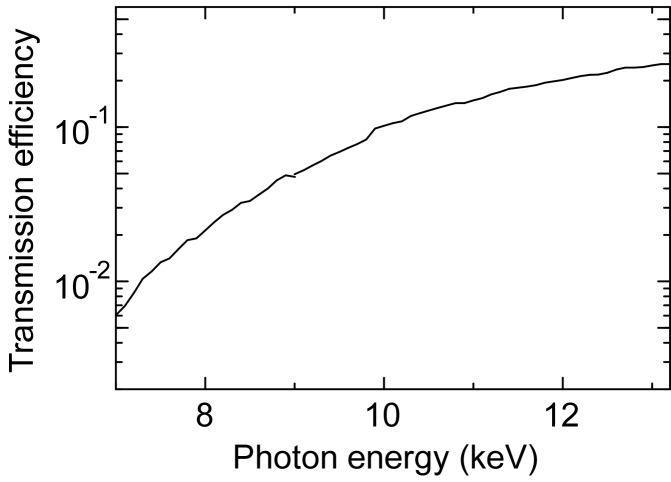
X-ray photon energy dependence of transmission efficiency for a 1.5 m-length fibre.

The transmission efficiency increases monotonically with increasing photon energies as shown in Fig. 3 ▶. In the photon energy range from 7.2 keV to 13.2 keV, a smooth energy dependence was observed without absorption resonance which exists in the soft X-ray region of 90–160 eV (Mazuritskiy, 2012 ▶). The high efficiency in the higher photon energy region is useful for application to hard-X-ray imaging. The low efficiency at the lower photon energies is dominant due to the increase of absorption at the wall, which is attributed to β in the refractive index (Sun & Ding, 2005 ▶). In the high-photon-energy region above 13.2 keV for which we did not measure the transmission efficiency, it has been suggested that the efficiency has a maximum and then decreases as the photon energy increases, as shown by Nakazawa (1983 ▶) for an X-ray guide tube for white X-rays.

### 4.3. Beam shifted area

**Figure 4:**
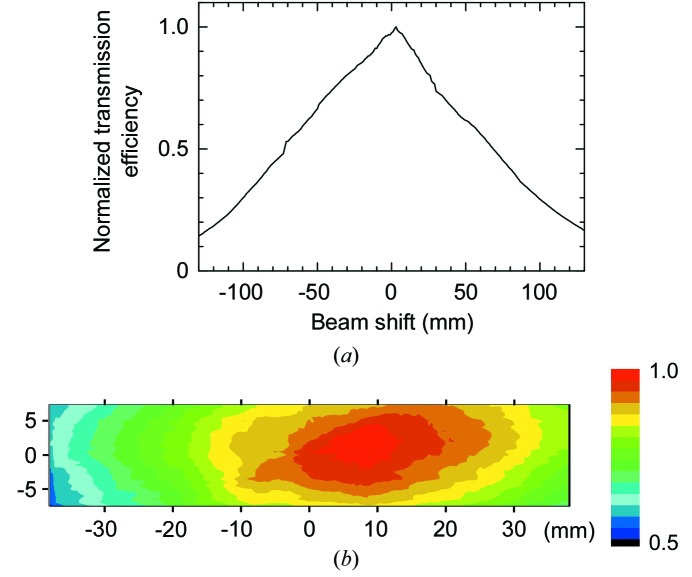
Performance of an X-ray beam axis scan with a 1.5 m-length fibre. (*a*) Horizontal shift dependence of normalized transmission efficiency for a photon energy of 12.4 keV. (*b*) Two-dimensional intensity distribution of beam scan for a 76 mm (horizontal) × 15 mm (vertical) area.

Fig. 4(*a*) ▶ shows the output beam intensity as a function of beam shift, *x*, in the horizontal direction. The available scan range is over ±75 mm with an intensity decrease of less than half of the peak. The range is consistent with the estimation given in Fig. 1(*b*) ▶. The achieved shift by 75 mm corresponds to mirror reflections by more than 43 pieces of SiO_2_ plate. The non-smooth change in intensity and the slightly asymmetric shape are due to the flexibility of the fibre. The reproducibility is also affected by the change in throughput due to the reproducibility of the whole curved shape of the fibre determining the beam trajectory for a shift. The deviation of the throughput efficiency for a shift is about 10% (peak-to-peak) in the present system. This requires normalized procedures for quantitative measurements. In order to achieve the high reproducibility in the efficiency, a mechanical system to fix the fibre trajectory should be developed in the future. A vertical axis shift was also performed in order to obtain an intensity area map, as shown in Fig. 4(*b*) ▶. The distribution in the vertical axis has a slightly narrower width than that in the horizontal axis due to the anisotropy caused by hanging the fibre with strings.

### 4.4. Output beam profile

**Figure 5:**
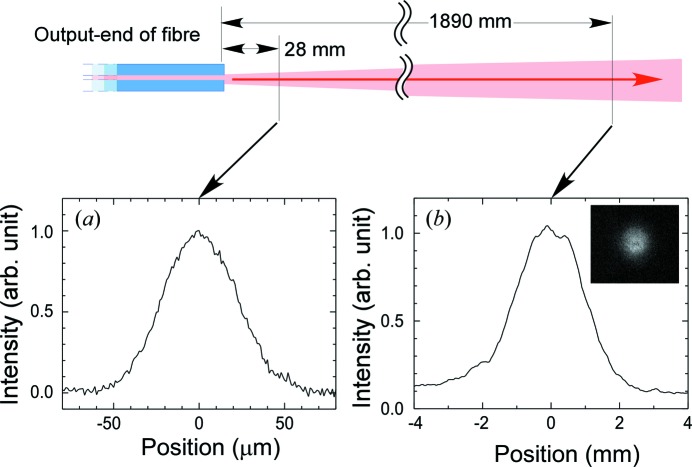
(*a*) Output beam profile at 28 mm downstream from the output end of the X-ray fibre, measured using a knife-edge scan in the horizontal direction. (*b*) Output beam profile in the horizontal direction observed 1890 mm downstream, with a flat-panel detector. The divergence of the beam is estimated to be 1.1 mrad from the profile. The inset shows the two-dimensional profile.

The output beam profiles in the horizontal direction at 28 mm and 1890 mm downstream from the output end of the fibre are shown in Figs. 5(*a*) and 5(*b*) ▶, respectively. The vertical profiles have almost the same distributions, as seen in the two-dimensional profile shown in the inset of Fig. 5(*b*) ▶. From the two beam profiles in Figs. 5(*a*) and 5(*b*) ▶ the beam divergence is estimated to be 1.1 mrad. Note that simple connection between fibres for extension is feasible, because the output beam divergence is smaller than the acceptance angle at the input end (∼1.6 mrad).

## 5. Demonstration of two-dimensional X-ray mapping

**Figure 6:**
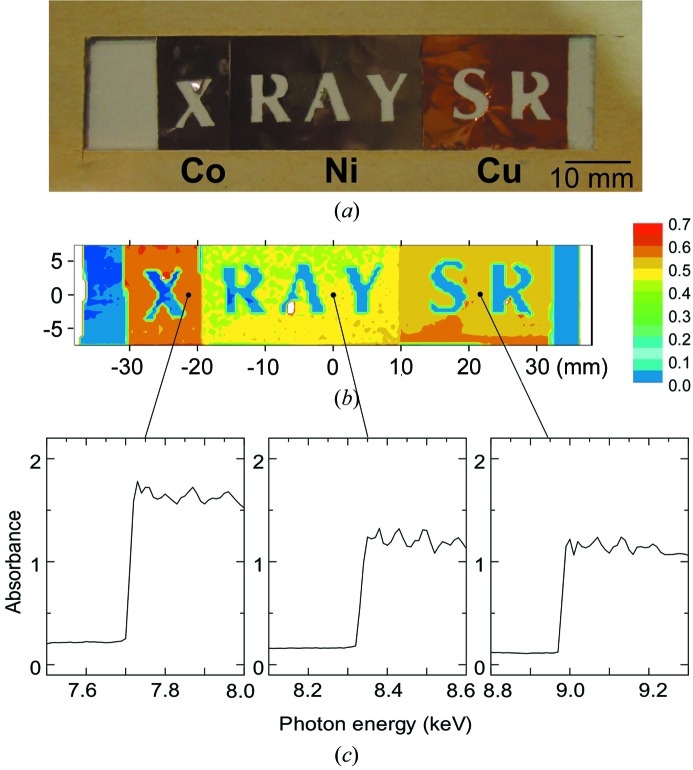
Wide-area X-ray mapping for a fixed test sample using a fibre scan. (*a*) Test sample composed of cobalt, nickel and copper films, of thickness 15 µm, 10 µm and 10 µm, respectively. (*b*) Absorbance map at a photon energy of 12.4 keV. The scan step and integration time were set to be 500 µm and 1 s, respectively, in order to save the measurement time, although the potential spatial resolution is 50 µm as obtained from Fig. 5(*a*) ▶. (*c*) Graphs showing X-ray absorption spectra at the positions indicated on the absorbance map.

As an application of the wide-area beam axis scan, we demonstrated absorption mapping for a fixed test sample with a size of 60 mm (horizontal) × 15 mm (vertical). The sample is composed of cobalt (15 µm-thick), copper (10 µm) and nickel (10 µm) films as shown in Fig. 6(*a*) ▶. It was positioned at 28 mm from the end of the fibre. Fig. 6(*b*) ▶ shows the absorbance distribution for 12.4 keV X-rays. The absorbance [log_10_(*I*_0_/*I*)] was obtained from the X-ray intensity (*I*) detected with a pin-photodiode which was normalized by the intensity (*I*_0_) with an ionization chamber located in front of the sample as shown in Fig. 2 ▶. The difference in absorbance by the area is clearly observed as well as the open letters. Fig. 6(*c*) ▶ shows the X-ray photon energy dependence of absorbance measured for the positions indicated in Fig. 6(*b*) ▶. The abrupt change in absorbance, indicating the *K*-edge of the element, identifies the ingredient elements to be cobalt, nickel and copper at the indicated positions.

## 6. Summary and perspective

X-ray beam trajectory control has been performed with a 1.5 m-long fibre whose bore and cladding diameters are 20 µm and 1.5 mm, respectively. The large cladding diameter maintains a moderate curvature which satisfies a shallow total reflection angle for X-rays. The observed transmission efficiency was more than 20% at a wavelength of around 0.1 nm. Using the fibre at a synchrotron radiation facility to control the beam trajectory, we demonstrated X-ray absorption mapping by scanning the beam for a fixed test sample. The obtained performance leads directly to advanced use: owing to the small divergence of the output beam it is possible to combine the fibre optics and waveguide optics to focus the beam to a few nanometres (Jark *et al.*, 2001 ▶) for high-resolution mapping, as well as the connection between fibres. For X-ray pulse timing control, a fibre with *l* = 1.5 m and *d* = 20 µm can achieve an optical delay of up to 6 ps, at the same position with an angle of 4°. This will enable us to perform jitter-free X-ray pump and X-ray probe experiments using a sub-picosecond pulsed X-ray beam (Inubushi *et al.*, 2012 ▶). On the other hand, an input coupler for the fibre should be developed for practical use to transport more X-ray beam flux, which may be improved by using focusing devices.
